# Breaking Barriers, Building Habits: Psychological Analysis of the Relationship Between Perceived Barriers, Financial Burden, and Social Support on Exercise Adherence Among Adults Aged 50 and Older in South Korea

**DOI:** 10.3390/healthcare13121469

**Published:** 2025-06-18

**Authors:** Suyoung Hwang, Eun-Surk Yi

**Affiliations:** Department of Exercise Rehabilitation & Welfare, Gachon University, Incheon 21936, Republic of Korea; harriett0059@gachon.ac.kr

**Keywords:** behavioral exercise adherence, constraint negotiation theory, perceived social support, financial burden, older adults

## Abstract

**Background/Objective:** Sustained exercise adherence among older adults is essential for healthy aging but remains challenging due to psychological, social, and economic barriers. This study aimed to investigate how perceived exercise barriers, financial burden, constraint negotiation mechanisms, and social support influence exercise adherence among adults aged 50 and older by integrating behavioral economics, constraint negotiation theory, and social cognitive theory. **Methods:** A cross-sectional survey was conducted with 1000 community-dwelling older adults in South Korea using a convenience sampling method. Participants were recruited from community centers, senior welfare facilities, and public health clinics in urban and suburban areas. Data collection was conducted between 11 January and 21 April 2024, using both online (Qualtrics) and offline (paper-based) surveys. Participants completed validated instruments measuring perceived exercise barriers, financial burden, constraint negotiation mechanisms (including financial management and social support mobilization), perceived social support, and behavioral exercise adherence. The final sample used for analysis included 974 individuals (mean age = 60.24 years, *SD* = 6.42). Structural Equation Modeling (SEM) was used to assess direct, mediating, and moderating effects. Additional exploratory analyses (ANOVA and *t*-tests) examined subgroup differences. **Results:** SEM results showed that perceived exercise barriers (β = –0.352, *p* < 0.001) and financial burden (β = –0.278, *p* < 0.001) were negatively associated with exercise adherence. Constraint negotiation mechanisms (β = 0.231, *p* < 0.001) and perceived social support (β = 0.198, *p* < 0.001) were positively associated. Mediation analyses revealed that constraint negotiation strategies partially mediated the relationships between perceived barriers and adherence (indirect β = 0.124) and between financial burden and adherence (indirect β = 0.112). Moderation analysis confirmed that social support buffered the negative effects of financial and psychological barriers. **Conclusions:** Exercise adherence in later life is shaped by the dynamic interplay of structural constraints, behavioral strategies, and social reinforcement. Interventions should combine financial support with socially embedded structures that promote behavioral planning and peer accountability to sustain long-term physical activity among older adults.

## 1. Introduction

The World Health Organization defines health not merely as the absence of disease or infirmity, but as a complete state of physical, mental, and social well-being [1]. This holistic understanding of health is especially critical in later life, when individuals are more likely to experience a confluence of challenges—including declining physical capacity, the accumulation of chronic illnesses, and increasing social isolation [2,3]. Maintaining health across all these dimensions becomes a central determinant of overall quality of life in aging populations. In this context, regular physical activity functions as a powerful health-promoting strategy, simultaneously supporting musculoskeletal function, emotional stability, cognitive preservation, and social engagement [4,5,6].

Despite widespread awareness of the benefits of physical activity, actual exercise participation among older adults remains strikingly low. According to the World Health Organization (WHO), an estimated 30–40% of adults aged 50 and over globally fail to meet recommended physical activity levels [7]—a deficit that contributes to widening health disparities and increasing chronic disease burdens. The implications extend beyond individual health outcomes, affecting public health expenditures, social cohesion, and the overall well-being of aging societies [8,9]. However, it is not merely the initiation of physical activity that contributes to healthy aging, but rather the sustained adherence to regular exercise routines over time. Consistent participation in physical activity is closely linked to maintaining functional independence, preventing age-related decline, and enhancing overall well-being in older adults [10]. Therefore, promoting exercise adherence is essential to achieving the benefits of healthy aging.

Previous studies have investigated various determinants of exercise adherence in older adults, with some focusing on psychological factors such as reduced motivation and self-efficacy [11,12,13], and others examining structural and environmental factors such as accessibility and financial limitations [14,15]. These studies have highlighted that a combination of internal barriers (e.g., health anxiety, low confidence) and external constraints (e.g., cost, limited access to exercise facilities, insufficient social support) jointly hinder regular physical activity among older adults. However, most existing research has analyzed these factors in isolation, without adequately exploring how they interact within an integrated theoretical model. This has resulted in a limited understanding of how psychological, structural, and environmental variables co-influence sustained exercise behavior [16,17].

To address this gap, the present study employs an integrative theoretical framework that draws from three complementary perspectives: behavioral economics, constraint negotiation theory, and social cognitive theory. Behavioral economics offers insights into why individuals often forgo long-term health benefits due to present bias and loss aversion, especially when facing immediate financial constraints [18,19]. For economically vulnerable older adults, even low-cost physical activity may be perceived as a “loss”, thereby reducing the likelihood of participation.

Constraint negotiation theory contributes a behavioral lens, emphasizing that individuals are not passive in the face of constraints but actively engage in strategies to maintain desired activities [20,21]. This includes financial reallocation and the mobilization of social support. In this study, social support mobilization is not treated merely as a contextual condition but as a behavioral strategy—an adaptive response that enables continued participation in the face of barriers.

Social cognitive theory serves as the core psychological foundation of the framework. It highlights the reciprocal interaction between self-efficacy, social modeling, and reinforcement in maintaining health behaviors [22]. Here, perceived social support is posited to not only facilitate behavior directly but also moderate the negative influence of psychological and financial barriers by boosting confidence, reducing isolation, and reinforcing adherence through social norms.

A key construct in this framework is behavioral exercise adherence, which this study defines as the frequency of physical activity and the intention to maintain it over time. This conceptualization moves beyond attitudinal measures to capture actual behavioral engagement, thereby addressing the frequently observed intention–behavior gap in physical activity research.

Accordingly, this study aims to empirically examine how exercise adherence among adults aged 50 and above is shaped by the interplay between perceived financial burden, exercise-related psychological barriers, and perceived social support. Specifically, the study seeks to:

- Identify key psychological and structural barriers to sustained physical activity;
- Investigate the mediating role of constraint-negotiation strategies—namely, financial management and social support mobilization;
- Test the moderating effect of perceived social support on the relationship between constraints and adherence.

By leveraging this multi-theoretical approach, the study aims to generate empirically grounded insights that inform the design of health promotion strategies that are economically feasible, psychologically informed, and socially embedded. Ultimately, this work contributes to the development of more equitable and sustainable physical activity interventions tailored to the needs of aging populations.

## 2. Theoretical Framework

To investigate the multidimensional determinants of behavioral exercise adherence in later life, this study integrates three complementary theoretical frameworks: behavioral economics, constraint negotiation theory (CNT), and social cognitive theory (SCT). Among them, SCT serves as the core psychological foundation, while behavioral economics and CNT offer explanatory mechanisms for structural and behavioral factors affecting adherence.

### 2.1. Behavioral Economics and Financial Determinants

Behavioral economics offers a powerful lens to understand why individuals may fail to act in alignment with long-term health interests. Foundational principles, such as present bias and loss aversion [23], and temporal discounting [24] explain why older adults, especially those under financial stress, may undervalue long-term exercise benefits in favor of short-term financial relief. For example, economic barriers like gym fees or transport costs may become salient deterrents, even when the health value of physical activity is fully recognized. Studies have shown that loss-framed incentives (e.g., forfeiting deposits if exercise is not completed) can enhance adherence more effectively than gain-framed strategies [25].

### 2.2. Constraint Negotiation Theory and Behavioral Adaptation

CNT emphasizes that individuals are not passive recipients of constraints but can actively negotiate them through behavioral strategies [26]. Particularly in older populations, constraints may include declining physical health, economic limitations, and reduced social interaction. Within this framework, individuals adopt constraint negotiation mechanisms—such as reallocating resources or modifying routines—to sustain meaningful participation. In this study, constraint negotiation mechanisms are operationalized through two subcomponents:

- Financial Management Strategies, such as budgeting for activity-related costs.
- Social support mobilization, including seeking exercise companions or joining peer-based programs.

Social support mobilization is conceptualized as a sub-strategy within the constraint negotiation domain, emphasizing its function as a behavioral coping mechanism rather than an independent construct. CNT is thus modeled as a mediator, explaining how perceived barriers are transformed into sustained behavior through proactive adaptation [27].

### 2.3. Social Cognitive Theory: The Core Psychological Lens

Bandura’s social cognitive theory (SCT) provides the study’s central psychological grounding [22]. Unlike traditional motivation theories focused solely on internal drivers, SCT posits a reciprocal determinism among personal beliefs, social environments, and behaviors. Critical constructs include self-efficacy, social reinforcement, and observational learning—all of which influence whether individuals initiate and maintain health-related routines.

In this model, perceived social support is treated as both a direct predictor and a moderator, buffering the negative effects of financial stress and psychological barriers. For example, older adults who engage in group exercise or receive encouragement from family and peers may be more confident and persistent in maintaining physical activity, even under constrained conditions [28]. This aligns with SCT’s emphasis on environmental enablers, social modeling, and accountability systems.

The theory also supports the inclusion of social support mobilization as a behavioral strategy embedded within broader negotiation efforts. This dual interpretation bridges SCT and CNT, revealing how perceived support is both a psychological state and an actionable strategy that enables continued engagement.

### 2.4. Integrated Model of Exercise Adherence

The integration of these three frameworks results in a cohesive and context-sensitive model of behavioral adherence in later life:

- Behavioral economics explains the decision-making biases and cost-sensitivity that hinder long-term exercise behavior [23,24,25].
- Constraint negotiation theory provides insight into adaptive mechanisms older adults use to manage perceived or real barriers [26,27].
- Social cognitive theory, as the anchoring lens, accounts for how social context, efficacy beliefs, and reinforcement sustain behavior over time [22,28].

Together, these theories enable a multi-layered understanding of why some older adults persist in physical activity despite economic or motivational challenges. The integrated framework not only supports empirical hypothesis testing but also offers actionable guidance for designing socially grounded, economically feasible, and psychologically empowering interventions for aging populations.

## 3. Hypotheses Development

This study draws on an integrated theoretical framework comprising social cognitive theory (SCT), constraint negotiation theory, and behavioral economics to examine the psychological, financial, and social determinants of exercise adherence among middle-aged and older adults.

According to SCT, health-related behaviors are shaped by a dynamic interplay between personal factors (e.g., beliefs, self-efficacy), social environments (e.g., modeling, support), and actual behavior [22]. Exercise adherence in later life often depends not only on individual intention but also on social reinforcement and perceived control. In particular, perceived exercise barriers such as lack of motivation, absence of support, or facility inaccessibility can diminish self-efficacy and outcome expectations, leading to lower adherence [29].

This leads to the first hypothesis:

**H1.**  *Perceived exercise barriers will negatively influence behavioral exercise adherence among middle-aged and older adults.*

To overcome these barriers, individuals often adopt constraint negotiation strategies—behavioral tactics that allow them to maintain desired activities despite structural or psychological constraints [26,27]. These strategies include financial reallocation (e.g., budgeting for exercise) and social support mobilization (e.g., finding exercise companions). Such proactive efforts are considered mediators that reduce the adverse impact of perceived barriers.

**H2.**  *Constraint negotiation mechanisms will mediate the relationship between perceived exercise barriers and behavioral exercise adherence.*

From the perspective of behavioral economics, older adults under financial stress may undervalue long-term health benefits and instead prioritize immediate financial needs [24]. This present bias, coupled with loss aversion [23], often leads to avoidance of health investments like gym memberships or structured exercise programs.

**H3.**  *Financial burden will negatively influence behavioral exercise adherence.*

Constraint negotiation also extends to social resource mobilization. Older adults may proactively seek companions or join community programs to offset psychological or structural barriers. Drawing again on SCT, social interactions can improve confidence, reinforce behavior, and normalize physical activity routines [28].

**H4.**  *Social support mobilization, as a form of constraint negotiation, will mediate the relationship between perceived exercise barriers and behavioral exercise adherence.*

Beyond its mediating function, perceived social support can serve as a moderating buffer against the negative effects of financial and psychological constraints. Previous studies have shown that social networks reduce the perceived weight of stressors, foster accountability, and improve adherence outcomes [30,31]. SCT posits that environments rich in reinforcement and role models facilitate sustained health behavior even when personal barriers remain.

**H5.**  *Perceived social support will moderate the negative relationship between financial burden and behavioral exercise adherence, in such a way that individuals with higher perceived support will show greater adherence despite financial constraints.*

**H6.**  *Perceived social support will moderate the negative relationship between perceived exercise barriers and behavioral exercise adherence, in such a way that individuals with stronger social networks will exhibit higher adherence despite such barriers.*

Together, these hypotheses form the empirical basis for testing a comprehensive model of exercise adherence in older adults. By incorporating both structural and psychological perspectives, the study seeks to offer theoretically grounded, policy-relevant insights into sustainable physical activity promotion in aging populations.

**Figure 1:**
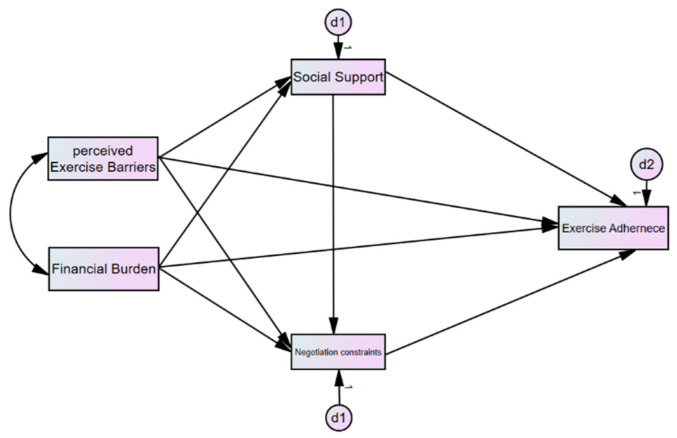
Conceptual framework illustrating hypothesized relationships among key variables.

Figure 1 presents the conceptual framework guiding this study, outlining the hypothesized relationships among perceived exercise barriers, financial burden, constraint negotiation strategies, perceived social support, and behavioral exercise adherence.

## 4. Methodology

### 4.1. Study Design

This study employed a cross-sectional survey design to examine the relationships among perceived exercise barriers, financial burden, constraint negotiation mechanisms, perceived social support, and behavioral exercise adherence in middle-aged and older adults. A cross-sectional approach is effective for identifying associations between key variables at a single time point, although it does not permit causal inference. Interpretations of the data are thus limited to correlational findings.

Quantitative analysis was conducted using Structural Equation Modeling (SEM), which allows for the simultaneous estimation of multiple direct, mediating, and moderating effects within a theoretically specified model. SEM was chosen to rigorously test the hypothesized relationships derived from social cognitive theory (SCT), constraint negotiation theory, and behavioral economics.

In addition to SEM, exploratory group comparisons were conducted to examine variations in exercise adherence across subgroups (e.g., economic status and exercise companionship). These were not pre-registered hypotheses but were included as theoretically meaningful supplements to enrich contextual interpretation.

### 4.2. Participants and Sampling

Participants included 1000 community-dwelling adults aged 50 and older who engaged in at least minimal levels of daily physical activity. Recruitment targeted individuals who regularly attended welfare centers, public health clinics, and senior citizen facilities in both urban and suburban areas.

A convenience sampling method was employed, and data were collected between 11 January and 21 April 2024, using both online (Qualtrics) and offline (paper-based) surveys to enhance accessibility and inclusivity. Offline data collection was conducted at senior welfare centers and public health institutions by trained research assistants. These assistants provided direct guidance and clarification to participants completing paper-based questionnaires, and responses were immediately collected on-site to ensure completeness and data accuracy.

To ensure methodological rigor and sampling accuracy, the online data collection process was monitored for duplication, incomplete responses, and inconsistent answering patterns through built-in Qualtrics validation logic and response-time filters.

The study also collaborated with EMBRAIN, South Korea’s largest online research panel provider with ISO 20252 certification [32]. EMBRAIN recruits participants from a verified nationwide panel of over 1.3 million adults, ensuring demographic diversity and response reliability. Online invitations were stratified by region and age, and participation was limited to unique verified IDs to avoid duplicate responses.

Importantly, participants were selected based on their report of engaging in regular physical activity at least once per week over the previous month. This inclusion criterion was intentional and aligns with the study’s central aim: to examine the psychological and structural mechanisms that support sustained exercise adherence, rather than initial adoption. Including sedentary or non-active individuals would have introduced factors related to exercise initiation, which falls outside the theoretical and empirical scope of the current study. Thus, by focusing on individuals who are already exercising to some extent, the study aimed to isolate the behavioral, financial, and social dynamics that enable or hinder ongoing participation.

Given the sampling frame, the study may overrepresent physically active and socially engaged older adults. To improve representativeness in future studies, stratified or random sampling methods are recommended—particularly to include mobility-limited or socially isolated individuals.

Table 1 summarizes the demographic characteristics of the respondents. While the total sample consisted of 1000 individuals, item-level responses ranged from 974 to 1000 due to minor missing data.

### 4.3. Data Collection Procedures

Data were collected via two modalities: an online survey administered through Qualtrics and a paper-based version distributed at community health and fitness centers. Trained research assistants facilitated on-site data collection to ensure clarity and completion.

Participants received a small incentive (monetary or fitness-related item) upon completing the survey. In-person participants were compensated immediately, while online participants received electronic rewards via email.

To ensure survey clarity and reliability, a pilot study (*n* = 50) was conducted before full-scale data collection. Based on feedback, several items were re-worded to improve readability and cultural relevance. Internal consistency for all final scales exceeded acceptable thresholds (Cronbach’s α ≥ 0.80).

## 5. Measurement Instrument

A structured questionnaire was developed to assess five constructs: perceived exercise barriers, financial burden, constraint negotiation mechanisms, perceived social support, and behavioral exercise adherence. All instruments were adapted from validated scales and revised for contextual relevance among older adults. Revisions were guided by a pilot test (N = 50) to enhance clarity, cultural appropriateness, and conceptual alignment.

Perceived exercise barriers were measured using a modified version of the Exercise Barriers Scale [33], with additional adaptation from later work tailored to older populations [34]. The construct included three subdimensions:

- Intrinsic barriers: e.g., low motivation, lack of confidence (4 items; α = 0.85);
- Interpersonal barriers: e.g., lack of social encouragement (3 items; α = 0.84);
- Structural barriers: e.g., cost and limited access (3 items; α = 0.87).

This construct informed Hypotheses H1 and H6.

Financial burden was assessed using a single item adapted from [35], assessing whether financial limitations prevented respondents from participating in physical activity. As this construct was operationalized with a single item, reliability indices such as Cronbach’s alpha were not computed. The item was selected based on prior validated use in health behavior research and was intended to reduce response burden while directly capturing perceived economic constraint.

Constraint negotiation mechanisms were measured using four items adapted from Hubbard & Mannell and Alexandris et al. [26,27], comprising two subcomponents:

- Financial Management Strategies (2 items; α = 0.82);
- Social support mobilization (2 items; α = 0.85).

Importantly, social support mobilization was treated as a behavioral subcomponent of constraint negotiation, not as an independent variable. These dimensions served to test H2 and H4.

Perceived social support was measured using the Multidimensional Scale of Perceived Social Support (MSPSS) [30], covering family, friends, and significant other domains (12 items; α = 0.90). This construct was used as both a predictor and a moderator (H5, H6).

Behavioral exercise adherence was operationalized using a modified version of the Godin Leisure-Time Exercise Questionnaire [36], including

Exercise frequency: weekly sessions (1 item; α = 0.83);

Exercise intention: future behavior prediction (2 items; α = 0.84).

This variable was used across all core hypotheses (H1–H6).

## 6. Statistical Analysis

All analyses were conducted using IBM SPSS Statistics 28 and AMOS 27. The analytic strategy followed a three-stage approach:

### 6.1. Descriptive Statistics and Preliminary Screening

Prior to statistical analysis, data screening procedures were performed to ensure accuracy and reliability. Of the 1000 initial cases, minor item-level missing data were addressed using listwise deletion, resulting in a final analytic sample of 974 complete cases.

Outliers were examined with both univariate (using standardized z-scores ± 3.29) and multivariate analyses (using Mahalanobis distance with *p* < 0.001); no significant outliers were found. The normality of continuous variables was assessed through skewness and kurtosis (within ±2.00 range), as well as histogram and Q–Q plot inspections. These evaluations confirmed that the assumptions of normal distribution were adequately met.

Means (M), standard deviations (SDs), and Pearson correlation coefficients (r) were calculated to examine bivariate relationships and detect multicollinearity.

### 6.2. Confirmatory Factor Analysis (CFA)

Model adequacy was assessed based on the following criteria:

- Composite Reliability (CR) ≥ 0.70
- Average Variance Extracted (AVE) ≥ 0.50
- Discriminant validity assessed via the Fornell–Larcker criterion [37]

### 6.3. Structural Equation Modeling (SEM)

SEM was used to assess direct, mediating, and moderating effects:

Mediation: the bootstrap method (5000 resamples) was applied to generate bias-corrected confidence intervals [38].

Moderation:

(a) Interaction term analysis was used to model multiplicative terms (e.g., social support × financial burden).
(b) Multi-group analysis (MGA) was performed across high vs. low support subgroups.

Measurement invariance was confirmed using configural, metric, and scalar tests (ΔCFI < 0.01), ensuring that group comparisons were valid and unbiased [39,40].

Model Fit was assessed using conventional criteria:

- CFI ≥ 0.90;
- TLI ≥ 0.90;
- RMSEA ≤ 0.08.

These thresholds follow Hu & Bentler and Hair et al. [41,42].

### 6.4. Exploratory Analyses

Two supplemental analyses were conducted:

- A one-way ANOVA to compare behavioral adherence across economic strata.
- An independent samples *t*-test to examine differences in adherence and perceived burden based on exercise companionship.

All tests were conducted on valid cases (*n* = 974), with listwise deletion applied to 26 participants with missing responses for key demographic variables. These exploratory results provided additional behavioral insights under practical conditions and reinforced the primary SEM outcomes.

## 7. Results

This section reports empirical findings from the study, covering descriptive statistics, bivariate correlations, Structural Equation Modeling (SEM), mediation and moderation analyses, and exploratory subgroup comparisons.

### 7.1. Correlation Analysis and Summary of Results

Full descriptive statistics, including raw means, standard deviations, and frequencies of key variables, are provided in Table 2. Descriptive statistics and Pearson correlations among the main constructs are presented in Table 3. Behavioral exercise adherence (*M* = 3.12, *SD* = 1.28) was significantly negatively correlated with perceived exercise barriers (r = –0.38, *p* < 0.001) and financial burden (r = –0.28, *p* < 0.001), while showing significant positive associations with constraint negotiation mechanisms (r = 0.23, *p* < 0.001) and perceived social support (r = 0.30, *p* < 0.001).

All correlation coefficients were below the multicollinearity threshold (r < 0.80), indicating acceptable discriminant validity for subsequent structural modeling.

### 7.2. Structural Equation Modeling (SEM) Results

The results from the SEM analysis are displayed in Table 4. All hypothesized direct and interaction paths were statistically significant and in the expected direction.

Perceived exercise barriers (β = –0.352, SE = 0.045, *p* < 0.001) and financial burden (β = −0.278, SE = 0.052, *p* < 0.001) had strong negative effects on behavioral exercise adherence.

Constraint negotiation mechanisms (β = 0.231, SE = 0.038, *p* < 0.001) and perceived social support (β = 0.198, SE = 0.041, *p* < 0.001) had significant positive effects on adherence.

Two interaction effects confirmed the moderating role of perceived social support:

- *Social support × Financial burden*: β = 0.129, SE = 0.037, *p* < 0.001
- *Social support × Perceived exercise barriers*: β = 0.114, SE = 0.034, *p* = 0.002

These results support Hypotheses H5 and H6, respectively.

### 7.3. Model Fit Indices

As shown in Table 5, the structural model exhibited excellent fit to the data:

χ^2^/df = 2.81 (acceptable: ≤3.00);

- CFI = 0.947 (≥0.90);
- TLI = 0.932 (≥0.90);
- RMSEA = 0.056 (≤0.08).

The fit thresholds follow established criteria [42].

### 7.4. Mediation Analysis

Bias-corrected bootstrapping (5000 resamples) was employed to test indirect effects. As shown in Table 6, two significant partial mediation pathways were identified:

Constraint negotiation mechanisms partially mediated the relationship between perceived exercise barriers and behavioral exercise adherence (indirect β = 0.124, 95% CI [0.086, 0.164]), supporting H2.

Social support mobilization, treated as a behavioral subcomponent of constraint negotiation, mediated the relationship between financial burden and exercise adherence (indirect β = 0.112, 95% CI [0.079, 0.148]), supporting H4.

These results suggest that older adults’ active coping strategies—such as reallocating finances or engaging in peer-based exercise—play a central role in mitigating structural and motivational barriers.

### 7.5. Group Differences in Exercise Adherence by Economic Status (ANOVA)

To further explore behavioral differences, a one-way ANOVA was conducted to examine variations in exercise adherence across five levels of self-reported economic status. As reported in Table 7, there were statistically significant group differences in both

- Exercise intention;
- Exercise frequency.

(F(4, 969) = 7.52, *p* < 0.001.)

As shown in Table 7, statistically significant group differences were found in:

- Exercise intention
- Exercise frequency

*F*(4, 969) = 7.52, *p* < 0.001

Tukey’s HSD post hoc tests indicated that participants in the very low-income group scored significantly lower in exercise adherence than those in the high- and very high-income groups (*p* < 0.05). However, the finding for the “very high” group should be interpreted with caution due to its small sample size (*n* = 5).

### 7.6. Effect of Social Support Mobilization (Exercise Companionship)

To assess the behavioral impact of social engagement, independent samples *t*-tests compared participants who exercised with a companion to those who did not.

The following is displayed in Table 8:

- Those with companions reported significantly higher adherence (*M* = 3.97, *SD* = 1.10) than those without (*M* = 3.45, *SD* = 1.22), (*t*(972) = 4.67, *p* < 0.001).
- Companion exercisers reported lower perceived financial burden (*M* = 3.15, *SD* = 1.08) than those without (*M* = 3.72, *SD* = 1.15).

These results underscore the economic buffering function of social support mobilization—demonstrating that perceived cost barriers are attenuated when exercise is shared with a partner or peer group.

## 8. Discussion

This study offers robust empirical evidence on how perceived exercise barriers, financial burden, constraint negotiation mechanisms, and perceived social support shape behavioral exercise adherence among adults aged 50 and older. Grounded in behavioral economics, constraint negotiation theory, and social cognitive theory (SCT), the findings elucidate the complex interplay between structural constraints, adaptive strategies, and social reinforcement in sustaining physical activity behavior later in life.

### 8.1. Financial Burden as a Structural Barrier

Consistent with H3, financial burden emerged as a significant negative predictor of adherence. This supports key behavioral economic principles such as present bias and loss aversion [23,24], which suggest that older adults are prone to prioritizing immediate financial relief over long-term health gains. Even relatively low-cost activities may be perceived as burdensome in the context of fixed incomes or competing expenditures, positioning affordability as a behavioral determinant rather than merely a contextual factor.

### 8.2. Perceived Barriers and Constraint Negotiation Strategies

Support for H1 and H2 demonstrates that perceived barriers—both internal and external—directly reduce adherence, while constraint negotiation mechanisms partially mediate this relationship. In line with constraint negotiation theory [26,27], older adults who engage in strategic planning—such as reallocating resources or mobilizing peer support—are better able to sustain exercise routines. Persistence, therefore, reflects not only motivation but also behavioral problem-solving and resource adaptability.

### 8.3. Social Support as Mediator, Moderator, and Behavioral Strategy

H4 through H6 confirm that perceived social support operates both as a mediator and a moderator in the relationship between constraints and adherence. Socially supported individuals reported higher adherence even under challenging economic or motivational circumstances. This substantiates SCT’s emphasis on modeling, reinforcement, and self-efficacy [22,28,30,31], suggesting that peer interaction and encouragement not only foster confidence but also reinforce consistent engagement.

Crucially, this study also reconceptualized social support mobilization as a behavioral strategy—a subcomponent of constraint negotiation mechanisms. Acts such as partnering for exercise or joining group-based programs exemplify SCT’s principle of reciprocal determinism, wherein the individual shapes and is shaped by their social environment. Thus, perceived support acts not only as an external facilitator but also as an internally enacted coping mechanism.

### 8.4. Socioeconomic and Social Participation Differences

Exploratory subgroup analysis revealed meaningful behavioral differences. The ANOVA results confirmed lower adherence in lower economic strata, while participants who exercised with companions demonstrated both higher adherence and lower financial burden perception. This aligns with Social Comparison Theory [43], which posits that peer norms can normalize and reinforce health behavior. Group exercise may serve as a psychological cost-sharing mechanism that distributes both perceived effort and resource strain.

### 8.5. Beyond Financial Incentives

Although this study did not test incentive-based interventions directly, the results raise important questions about their long-term effectiveness. As previous research suggests, financial incentives often fail to maintain behavioral gains once removed [44]. The findings here support a more hybrid approach, combining modest economic incentives with behavioral planning tools and socially embedded accountability systems.

### 8.6. Broader Implications: Policy Recommendations and Community Applications

These findings have direct implications for the design of public health programs and aging-related interventions. First, community-based exercise initiatives should be tailored to account for not only physical limitations but also psychological and economic constraints, which were shown to significantly influence adherence. Policy designers should consider integrating low-cost or subsidized fitness opportunities within existing welfare infrastructures (e.g., public health clinics, senior welfare centers), especially in low-income districts.

Second, social support was found to buffer both motivational and financial barriers, suggesting that exercise programs that foster peer-based participation, group dynamics, and buddy systems may increase persistence. These can be facilitated through local governments or NGO-led programs and could target socially isolated individuals.

Furthermore, the reconceptualization of social support mobilization as a proactive behavioral strategy underscores the need to shift from a deficit-based model (“lack of support”) to a capacity-building approach where older adults are empowered to seek and maintain support networks.

Finally, these findings lend support to the development of hybrid interventions that integrate behavioral economics (nudging techniques), social reinforcement, and financial flexibility. For example, opt-out systems for community classes or default scheduling may increase participation by mitigating present bias. To operationalize these strategies effectively, we recommend combining. Targeted financial support with peer accountability mechanisms; Community-based exercise opportunities that activate social reinforcement; Behavioral planning tools such as budgeting aids and partner-matching systems.

These multidimensional approaches reflect the biopsychosocial model and address the psychological, economic, and relational determinants of exercise adherence in older populations.

### 8.7. Limitations and Future Research Directions

Despite the strengths of this study, several limitations should be acknowledged. First, participants were purposefully selected based on their engagement in regular physical activity at least once per week during the previous month. This inclusion criterion aligns with the central aim of the study—to explore psychological and structural mechanisms underpinning sustained exercise adherence, not the initial adoption of physical activity. Including sedentary or non-active individuals could have introduced confounding factors related to motivational deficits or health constraints unrelated to long-term behavioral maintenance. Thus, this selection strategy enabled a more focused examination of ongoing behavioral dynamics.

Nevertheless, the exclusive inclusion of physically active older adults, many of whom were recruited from community centers or welfare facilities, may limit the generalizability of the findings. Specifically, the study may underrepresent socially isolated or mobility-limited individuals who experience different types or intensities of exercise barriers. Future studies should consider employing stratified random sampling to include a more representative cross-section of the aging population, particularly those facing more severe health or environmental limitations.

## 9. Conclusions

This study investigated the multifaceted determinants of behavioral exercise adherence among adults aged 50 and older by integrating theoretical insights from behavioral economics, constraint negotiation theory, and social cognitive theory (SCT). Empirical findings confirmed that perceived exercise barriers and financial burden significantly hinder sustained physical activity engagement, while constraint negotiation mechanisms and perceived social support act as critical facilitators.

A central contribution of this study is its reconceptualization of adherence not merely as a motivational outcome, but as the result of strategic adaptation to structural and psychological constraints. Older adults who engaged in behavioral planning—such as managing exercise-related expenses or mobilizing peer companionship—demonstrated greater resilience in maintaining regular activity. Furthermore, social support was not only shown to influence behavior directly but also to moderate and mediate the impact of adverse financial and motivational conditions, in line with SCT’s emphasis on self-efficacy, modeling, and reinforcement.

From a behavioral economics perspective, the findings affirm the significance of present bias and loss aversion in shaping exercise decisions. Financial burden operates both directly and indirectly to limit participation, particularly when perceived as a potential “loss.” Yet, social and behavioral strategies, particularly those rooted in communal support, were found to mitigate these effects, providing pathways to behavioral sustainability.

Theoretically, this study demonstrates the value of integrating SCT with constraint negotiation and economic models to better understand behavioral persistence in aging populations. Practically, the findings highlight the limitations of financial incentives and motivational messaging alone. Interventions that combine financial tools, social reinforcement, and behavioral planning—delivered in accessible, relational contexts—are likely to yield more equitable and enduring outcomes.

From a policy standpoint, exercise promotion should prioritize community-based infrastructure that embeds relational and behavioral support. This includes subsidized fitness programs in public spaces, socially enriched digital health tools, and systems that reward social engagement. These should be seen not as optional adjuncts but as core components of public health strategies for aging societies.

Despite the strengths of this study, limitations remain. The cross-sectional design restricts causal interpretation, and reliance on self-reported data may introduce bias. Longitudinal and qualitative studies are needed to track how negotiation strategies evolve and to uncover deeper insights into the lived experiences of behavioral adherence across diverse economic and cultural settings.

Ultimately, as global populations continue to age, understanding how older adults sustain physical activity requires a shift from individual-centric models toward socially embedded and behaviorally informed frameworks. This study offers a theoretically grounded and empirically supported roadmap for designing interventions that do not merely initiate behavioral change but also maintain it in the face of real-world constraints.

## Figures and Tables

**Table 1:** Demographic characteristics of respondents by survey mode (*n* = 974).

Variable	Categories	Online (*n* = 610)	%	Offline (*n* = 364)	%
Gender	Male	308	50.5	188	51.6
	Female	302	49.5	176	48.4
Age	50–54	212	34.8	135	37.1
	55–59	121	19.8	68	18.7
	60–64	160	26.2	101	27.7
	65+	117	19.2	60	16.5
Type of Exercise	Aerobic	485	79.5	279	76.6
	Strength training	20	3.3	12	3.3
	Mixed	105	17.2	73	20.1
Exercise Frequency	Less than once per week	262	43.0	159	43.7
	Twice per week	125	20.5	72	19.8
	Three times per week	84	13.8	56	15.4
	Four times per week	44	7.2	26	7.1
	Five or more times per week	95	15.6	51	14.0
Economic Status	Very high	3	0.5	2	0.5
	High	90	14.8	56	15.4
	Middle	352	57.7	204	56.0
	Low	138	22.6	81	22.3
	Very low	27	4.4	21	5.8
Education Level	Middle school or below	10	1.6	9	2.5
	High school	139	22.8	94	25.8
	College	375	61.5	216	59.3
	Graduate school	86	14.1	45	12.4

Note: Percentages are calculated within each survey method group. Minor rounding may result in totals not equaling 100%.

**Table 2:** Full descriptive statistics: means, standard deviations, and frequencies of study variables (*n* = 974).

Variable	Sub-Dimension	*n*	*M*	*SD*
Perceived Exercise Barriers	Intrinsic Barriers	974	2.74	0.92
	Interpersonal Barriers	974	2.65	0.88
	Structural Barriers	974	2.95	0.90
Financial Constraints (Financial Burden)	Single Item	974	3.44	1.10
Constraint Negotiation Mechanisms	Financial Management	974	3.55	0.85
	Social Support Mobilization	974	3.78	0.89
Social Support	Family	974	4.20	0.75
	Friends	974	4.15	0.78
	Significant Others	974	4.30	0.72
Exercise Adherence	Exercise Frequency	974	3.12	1.28
	Exercise Intention	974	3.80	0.92

**Table 3:** Pearson correlations among core constructs with means and standard deviations (*n* = 974).

Variable	*M*	*SD*	1	2	3	4	5
Exercise Adherence	3.12	1.28	—				
Perceived Exercise Barriers	2.78	1.12	−0.38 **	—			
Financial Constraints (Financial Burden)	3.5	1.28	−0.28 **	0.14 **	—		
Constraint Negotiation Mechanisms	3.22	1.14	0.23 **	−0.25 **	−0.18 **	—	
Social Support	3.33	1.18	0.30 **	−0.19 **	−0.22 **	0.27 **	—

Note: All values are Pearson’s r coefficients. ** *p* < 0.01 (2-tailed).

**Table 4:** Structural Equation Modeling (SEM) results.

Hypothesis	Path	Standardized β	S.E.	t-Value	*p*-Value	Result
H1	Perceived Exercise Barriers → Behavioral Exercise Adherence	−0.352	0.045	−7.82	*p* < 0.001	Supported
H2	Constraint Negotiation Mechanisms → Behavioral Exercise Adherence	0.231	0.038	6.21	*p* < 0.001	Supported
H3	Financial Constraints (Financial Burden) → Behavioral Exercise Adherence	−0.278	0.052	−5.34	*p* < 0.001	Supported
H4	Perceived Social Support → Behavioral Exercise Adherence	0.198	0.041	4.83	*p* < 0.001	Supported
H5	Social Support × Financial Burden → Behavioral Exercise Adherence	0.129	0.037	3.48	*p* < 0.001	Supported
H6	Social Support × Perceived Exercise Barriers → Exercise Adherence	0.114	0.034	3.02	*p* = 0.002	Supported

**Table 5:** Model fit indices.

Model Fit Index	Threshold	Result	Fit Status
Chi-square divided by degrees of freedom (χ^2^/df)	≤3.0	2.81	Good Fit
Comparative Fit Index (CFI)	≥0.90	0.947	Good Fit
Tucker–Lewis Index (TLI)	≥0.90	0.932	Good Fit
Root Mean Square Error of Approximation (RMSEA)	≤0.08	0.056	Good Fit

Note: All fit indices meet or exceed recommended thresholds [41].

**Table 6:** Mediation analysis results.

Mediation Path	Direct Effect (β)	Indirect Effect (β)	95% CI (Lower–Upper)	Total Effect (β)	Result
Perceived Exercise Barriers → Constraint Negotiation Mechanisms → Behavioral Exercise Adherence	−0.352	0.124	(0.086–0.164)	−0.228	Partial Mediation
Financial Constraints (Financial Burden) → Social Support Mobilization (as CNM) → Behavioral Exercise Adherence	−0.278	0.112	(0.079–0.148)	−0.166	Partial Mediation

Note: Bootstrapping with 5000 resamples. CI = Confidence Interval. All indirect effects significant at *p* < 0.05.

**Table 7:** Differences in exercise adherence by economic status (ANOVA results).

Economic Status	N	Exercise Intention (*M*, *SD*)	Exercise Frequency (*M*, *SD*)	F-Value (*df* = 4, 969)	*p*-Value	Tukey’s HSD (*p* < 0.05)
very low	45	3.02 (1.21)	2.79 (1.35)	7.52	*p* < 0.001	Significantly lower than high and very high
low	220	3.28 (1.17)	3.01 (1.32)			
middle	556	3.69 (1.12)	3.15 (1.27)			
high	148	3.98 (1.09)	3.34 (1.22)			
very high	5	4.21 (0.98)	3.56 (1.18)			

Note: Exercise intention and frequency significantly differed by economic status (*F*(4, 969) = 7.52, *p* < 0.001). Tukey’s HSD post hoc test revealed that the “very low” group had significantly lower scores than the “high” and “very high” groups. Interpret results for “very high” group with caution due to small sample size (*n* = 5).

**Table 8:** Group differences in exercise adherence and financial burden by social support mobilization (*n* = 974).

Condition	N	Exercise Adherence (*M*, *SD*)	Financial Burden Perception (*M*, *SD*)	Mean Difference	t-Value (*df* = 998)	*p*-Value
With Companion	506	3.97 (1.10)	3.15 (1.08)	+0.52	4.67	*p* < 0.001
Without Companion	468	3.45 (1.22)	3.72 (1.15)			

## Data Availability

The raw data supporting the conclusions of this article will be made available by the authors upon reasonable request.
